# Coping with drug shortages: A study of government-enterprise option cooperation stockpiling strategies for drugs in shortage considering API surrogate stockpiling subsidies

**DOI:** 10.1371/journal.pone.0305383

**Published:** 2024-07-02

**Authors:** Yipeng Lan, Chenlu Meng, Lihua Sun, Zhe Huang

**Affiliations:** 1 School of Business Administration, Shenyang Pharmaceutical University, Shenyang, China; 2 Institute of Drug Regulatory Science, Shenyang Pharmaceutical University, Shenyang, China; Ladoke Akintola University of Technology Teaching Hospital: LAUTECH Teaching Hospital, NIGERIA

## Abstract

Drug shortage is a global problem, and the development of government-enterprise cooperative stockpiles of drugs in shortage, combining physical and production capacity, has become one of the most important means of coping with drug shortages. However, existing studies have tended to overlook the fact that shortages of Active Pharmaceutical Ingredients (APIs) have become an important constraint on production capacity stockpiling and that the lack of incentives and provisions for coordination of benefits have led to a double marginal effect of joint stockpiling by government and enterprises of drugs in shortage. Accordingly, this study introduced the option contract to the drug supply system composed of government and pharmaceutical enterprises and used the subsidy of API storage in lieu as an important initiative to incentivize the reserve of APIs, to construct a model of shortage drug reserve under the government’s leadership. This study aims to improve the effect of government-enterprise joint stockpiling of drugs in shortage, which is of great theoretical and practical significance. According to the classification of production license types of pharmaceutical enterprises, this study established a three-level supply chain decentralized decision-making model consisting of the government, formulation enterprises, and API enterprises, and a two-level supply chain centralized decision-making model consisting of the government and API Formulation (API-F) integrated enterprises, respectively. By solving the inverse order derivation, the government-enterprise option cooperation conditions and optimal decision-making strategy were derived. The study results showed that: (i) The addition of enterprise API stockpiling mode can help the government conventional reserves, and enterprise production capacity reserves, broaden the way of drug reserves, and improve the effect of government-enterprise option cooperation; (ii) when the probability of drug shortages is high, the government should prefer the cooperation of API-F integrated enterprises, which is conducive to reducing intermediate links and government costs and improving the supply responsiveness to shortages of medicines; (iii) Setting appropriate government subsidies for API storage can incentivize enterprises to stockpile APIs and improve drug production capacity and physical supply response capability. This study took the problem of socialized stockpiling of drugs in shortage as an entry point and explored the problems and solution strategies in the government-enterprise cooperative stockpiling of drugs in shortage, which not only made some theoretical contributions to the application of options contract in the government-enterprise cooperative stockpiling of drugs in shortage but also provided new ideas and theoretical basis for the improvement of the stockpiling work of drugs in shortage.

## 1 Introduction

The World Health Organization (WHO) has identified drug shortage as a worldwide problem [1]. Both developed and developing countries are facing drug shortages that threaten the health and quality of life of their citizens [2–6]. As the world’s largest developing country, China faces a serious shortage of medicines. According to survey data, 828 Chinese public hospitals reported 12,170 drug shortages of different specifications during 2019–2021 [6]. Ensuring a stable supply of medicines is not only an important task to meet the health needs of the people but also an important manifestation of building a strong pharmaceutical country. As the main body responsible for guaranteeing the supply of medicines, the Chinese government attaches great importance to the management and prevention of drug shortages and guarantees a stable supply of medicines by clarifying and reinforcing the responsibilities of all stakeholders in preventing drug shortages and giving full play to their respective monitoring and early-warning roles [7, 8]. More and more studies and practices have shown that adequate drug stockpiling is an important means of preventing drug shortages and ensuring a stable supply of drugs [9–13].

Since 2017, the Chinese government has issued several guidance documents on stockpiling of drugs in shortage [14–16]. Among them, a special chapter on drug stockpiling and supply was provided in the revised Drug Administration Law of the People’s Republic of China in 2019 [17]. This was the first time that China raised the regulation of shortage drug stockpiling and the strengthening of drug supply guarantee to the legal level. While the form of stockpiling of drugs in shortage, for which the government is solely responsible, ensures the timeliness of the supply of drugs, it also puts enormous financial pressure on the government. For example, some scholars have suggested that relying solely on the government to stockpile medical supplies will not only increase the construction and operating costs of the stockpile but also add to the costs of purchasing and managing medical supplies [18, 19]. Zhang et al. also suggested that separate government stockpiles of medical supplies would be at risk of shortages or surpluses, creating pressure on government inventories [20]. In this regard, multi-party stockpiling cooperation has become an inevitable choice for the government to improve its ability to guarantee the supply of medicines. Wang believed that the cost of drug reserves could be reduced by taking advantage of the scale of pharmaceutical enterprises’ reserves [21]; Hu believed that the efficient production capacity and turnover capacity of enterprises could be utilized to rotate and update the reserve drugs and improve the level of drug supply guarantee [22]. Although China in the successive release of the National Pharmaceutical Stockpile Management Measures and other documents has emphasized the need for government departments to contact pharmaceutical enterprises to carry out the combination of physical and production capacity of drug reserves [23–25]. Moreover, China recognized six centralized production bases for shortage drugs to promote the production of clinically shortage-prone APIs and formulations [26, 27]. However, from the Notice on Strengthening the Monitoring of Production Reserves of Drugs in Shortage and Selected Drugs in the Centralized Purchase of Drugs by State Organizations, jointly issued by the Ministry of Industry and Information Technology, the National Health Commission, and other departments in August 2022, it is shown that there are still 980 kinds of formulations and 256 kinds of APIs in shortage in varying degrees, and the situation is still serious [28]. The main reason for this is that the above policies are mainly based on normative requirements, without detailed rules for cooperation, and with fewer clear incentives and benefit coordination provisions [29, 30].

The reasons for this are mainly due to the fact that the above policy does not set out detailed details of cooperation, and also lacks clear incentives and provisions for harmonization of interests. This phenomenon is more common in areas such as drug supply and new energy development, such as Yin et al., who suggested that the lagging development of new energy equipment in rural areas has led to a large gap between the goals of rural modernization encouraged by the policy. The contrast between the policy objectives and the real effect just shows that there are conflicts of interest and other problems in the process of policy implementation, which the industry needs to be urgently resolved [31]. To better manage and coordinate the government-enterprise stockpile model, in recent years, the industry has commonly utilized the option contract theory to help government-enterprises develop rational decision-making plans for emergency medical supplies to ensure reliable supply, maintain stable prices, and satisfy uncertain demand [32–34]. The option contract is an economics program that is often used in supply chain management for trading parties to enter into uncertain demand product supply due to its characteristics such as risk management, flexibility, and no need to physically hold the asset [35, 36]. For example, Hu verified the effectiveness of option contracts in the supply chain coordination process under demand uncertainty [37]; Aghajani et al. used option contracts to derive the optimal production and purchasing quantities for supply chain decision makers under output and demand uncertainty [38]; Liu et al. suggested that option contracts can increase the level of joint government-enterprise physical and production capacity stockpiles of emergency supplies and improve the response level of disaster relief [39]. However, while existing research confirms that option pacts can coordinate government-enterprise cooperation to carry out physical and production capacity stockpiling of emergency supplies, drugs in shortage belong to special emergency supplies, and the primary prerequisite for promoting the security of supply of medicines through enterprise production capacity stockpiling is an adequate stockpile of APIs [40, 41]. The current shortage of APIs has become one of the main reasons for drug shortages in China, which is contrary to the reality of China as the world’s second-largest producer of APIs and the first-largest exporter of APIs [4, 5, 42].Various realities suggest that strengthening the API stockpile is the best way to realize the physical and production capacity of pharmaceuticals. Therefore, there is an urgent need to incorporate the mechanism and strategy of API stockpiling into the study and to further improve the incentive mechanism for drug stockpiling, to more fully utilize the advantages and effects of joint stockpiling by government and enterprises of drugs in shortage.

Given this, this study introduced the option contract to the drug supply system composed of the government and pharmaceutical companies, and used the API stockpile subsidy as an important initiative for the government to incentivize companies to carry out API stockpiling, to construct a model of government-led stockpiling of shortages of pharmaceuticals. According to the type of enterprise production licenses, this study established a three-level supply chain decentralized decision-making model consisting of the government, formulation enterprises, and API enterprises, and a two-level supply chain centralized decision-making model consisting of the government and API-Formulation (API-F) integrated enterprises, respectively. Through the model derivation and solution, the optimal reserve strategy of the government and enterprises and their respective costs and profits were derived, and a comparison and sensitivity analysis was made with the government’s separate reserve mode, to provide a guiding basis for the establishment of regularized cooperation between the government and enterprises, and better improve the efficiency of the reserve of shortages of medicines and the ability of supply guarantee.

This study has the following theoretical and practical significance: (i) It constructs a joint stockpiling model of government and enterprises in drugs in shortage based on option contracts, and makes a theoretical contribution to the application of option contracts in the cooperative stockpiling of government and enterprises in drugs in shortage. (ii) It explores the form of stockpiling of drugs in shortage and the means of subsidizing the stockpiling of APIs, which is conducive to improving the rules and incentives for the stockpiling of drugs in collaboration with the government and enterprises. (iii) It provides a guiding basis for the establishment of regularized government-enterprise cooperation on drugs in shortage, to improve better the efficiency of stockpiling drugs in shortage and the ability to guarantee their supply.

The rest of the paper is organized as follows: Section 2 is the Literature review and theoretical foundations, Section 3 describes the problem and assumptions, Section 4 is the benchmark model, Section 5 presents the model construction and solution, Section 6 is the comparison of equilibrium results, Section 7 is the numerical analysis and discussion, and Section 8 is the conclusions and future prospects.

## 2 Literature review and theoretical foundations

### 2.1 Literature review

In recent years, supply assurance of drugs in shortage has been a research hotspot in supply chain management. However, research on the issue of joint government-enterprise stockpiling of drugs in shortage considering government subsidies has not yet been explored. However, we can see research results in the fields of supply management of drugs in shortage, emergency stockpile management, supply chain contract model, and government subsidies that are related to this study.

The supply assurance of drugs in shortage is a major livelihood project, which has received great attention from government departments and academics. Through research or semi-structured interviews with stakeholders in the drug supply chain, some scholars have learned that the reasons for drug shortages include shortages or monopolization of APIs [43, 44], low transaction prices or difficulties in manufacturing [45], environmental protection, and regulatory impacts [10, 46, 47], with shortages of APIs being one of the most important reasons [48]. Thus, Tucker and Lee suggested that the problem of drug shortages can only be alleviated by improving stakeholder communication and coordination [49, 50]. Liu, Shi, and Dong proposed the need to be proactive instead of reactive by designing different prediction models for drug shortage risk and actively carrying out pre-emptive countermeasures for drug shortage [51–53]. Tucker proposed a pharmaceutical supply chain model with improvements in terms of configuration, disruption risk, and recovery speed to reduce the probability of drug shortage [54]. Moosivand prioritized drug shortage responses through a multi-criteria decision-making approach, suggesting the need to create robust drug inventories to enhance drug supply chain resilience [55]. While Abu Zwaida and Dewi have argued that storage cost and shortage risk can be effectively reduced by designing drug demand forecasting models [56, 57]. Paul and Saedi used deep learning models and stochastic models, respectively, to find the optimal inventory strategy for medicines to reduce the impact of supply chain disruptions on patients’ access to medicines [58, 59]. Thus, it can be seen that improving the level of drug stockpiles is widely recognized as an effective measure of ex-ante management to cope with drug shortages. However, the above studies mainly carry out drug storage research from the government side and do not apply to the current situation of socialized stockpiling of drug shortages encouraged by the state, resulting in a lack of applicability and expandability of their research.

Considering that the shortage of medicines belongs to special emergency supplies, the research results of the quantitative approach to emergency supplies stockpiling provide an important reference value for this study [60, 61]. For example, Wang conducted a study on the problems exposed by the emergency medical material stockpile and supply system in China, the United States, and Australia during the COVID-19 Pneumonia Epidemic, and proposed to improve the coordination mechanism of the government-enterprise cooperation stockpile of emergency materials [62]. Meng, Hou, and Pan also suggested that government-enterprise cooperation in emergency stockpiling can improve rescue efficiency and reduce government inventory risk and management costs, but the enterprise inventory capacity and government coordination mechanism have a significant impact on the optimal stockpile allocation strategy [63–65]. Zhang then explored the specific conditions and influencing factors for realizing government-enterprise cooperation by establishing a tripartite evolutionary game model of the government, enterprises, and society, and proposed the need to design a flexible procurement strategy to improve supply chain resilience [66]. In contrast, multi-party cooperation to promote policy implementation has been widely recognized, for example, Yin et al. suggested that the development of high-end agricultural machinery and equipment can be promoted through joint research by scientific research institutions and equipment manufacturing enterprises. This concept of cooperation is an important reference for the cooperation between the government and enterprises to carry out drug stockpiling in this study [67].

To further improve the coordination of interests in government-enterprise cooperation, some scholars, from the perspective of supply chain, have proposed to establish government-enterprise cooperation in utilizing supply chain contracts. For example, Inderfurth considered that the double marginalization effect hindered the coordination of supply chain interests and solved the supply chain coordination problem under stochastic demand by using a risk-sharing contract [68]. Rahimi-Ghahroodi designed a gain-sharing cooperation model using Stackelberg game theory as a way to improve supply chain members’ profits in emergency procurement [69]. Zhao studied the procurement of materials under the quantity flexibility contract and concluded that flexible procurement can reduce the retailer’s reserve cost [70]. While more scholars chose to utilize option contracts to conduct research, mainly because option contracts can increase the buyer’s flexibility to cope with the uncertain demand in the product market [71], and the option itself has a price discovery and risk aversion function [72, 73]. For example, Sun compared three emergency material procurement models, the option model, the pre-disaster order model, and the post-disaster procurement model, and the results showed that the option contract is more suitable for public sector emergency material procurement [74]. Fathalikhani’s study pointed out that option contracts can satisfy the objectives of improving social welfare maximization and budgetary consumption minimization of the government [75]. Liu and Wang also demonstrated that option contracts can satisfy the immediate post-disaster purchase condition and achieve Pareto improvements by coordinating the relief supply chain [76, 77].

Meanwhile, some scholars have further researched the details of cooperation in the option contract, such as the issue of production capacity pre-stocking, incentives, and so on. John studied the problem of vaccine procurement during an epidemic outbreak and proposed a flexible procurement model that combines advanced physical procurement, production capacity stockpile, and spot market purchase [78]. Wu argued that manufacturers can choose between production capacity stockpiles or spot market purchases based on whether market demand is certain or not and whether the wholesale price is fixed or not [79]. While Gao proposed that due to poor information sharing, enterprises are prone to emotional burnout, and the government subsidy mechanism can effectively improve the enthusiasm for enterprise cooperation [80]. Xiao and Wei indicated that emergency medical supplies reserve has public welfare, if the lack of incentive policies, enterprises are prone to violations or speculative behavior, resulting in insufficient capacity, thus proposing the incentive model of production capacity reserve to guide the government-enterprise cooperation to be carried out smoothly [81, 82].

The above findings laid the foundation for further analysis in this study, but the literature review revealed three shortcomings in the existing research. First, few scholars have conducted quantitative research in studying the problem of social stockpiling of drugs in shortage, and there is a lack of consideration of the issue of coordination of interests in government-enterprise cooperation. Secondly, although the existing research on option contracts has confirmed its advantages in coordinating the interests of government and enterprises in emergency stockpiling, there has not yet been any consideration for the special characteristics of shortage drugs, resulting in limited guidance for practice. Finally, almost all studies ignore the fact that the shortage of APIs is an important factor contributing to drug shortages, yet China is a major producer and exporter of APIs. Out of this reality, this paper innovatively proposes that strengthening the reserve of APIs is the key to enhancing the reserve of pharmaceutical production capacity. Based on this, this study takes shortage drugs, a special kind of emergency medical supplies, as the research object, and investigates how to improve the government-enterprise collaborative drug stockpiling work and improve the level of drug stockpiling by designing a reasonable option agreement. In this paper, we add the consideration that the government sets the API stockpile subsidy to incentivize the API reserve in the study, construct the coordination model of the shortage drug stockpile in the cooperation between government and enterprises, and analyze in depth the influence of the drug shortage probability, the option execution price, and the API stockpile subsidy on the stockpile decision of the government and enterprises. The research in this paper is a useful supplement to the option contract in the field of emergency supply chain.

### 2.2 Theoretical foundations

The main theories utilized in this study include the emergency stockpile theory and the option contract theory.

Although the drug is in shortage as a special emergency material, this study needs to use the emergency material stockpile theory to support it. The theory of emergency stockpile mainly stipulates the way of drug stockpile and the main body of stockpile. The main types of stockpiles include physical stockpiles and stockpiles of production capacity [22, 30]. Physical stockpiles are stored in physical form in warehouses and can be called upon whenever needed. Physical stockpiles are the main source of materials for the initial response to emergencies. They are important for safeguarding the needs of life, controlling the development of events, and saving lives. The production capacity stockpile is a way of reserving emergency supplies called the production capacity stockpile for those enterprises that are capable of producing supplies by signing a contract to ensure that they can rapidly produce supplies by the requirements of the contract after an emergency. In terms of reserve objects, it includes the government and production enterprises [66, 70]. Government stockpile means that the government will store some important materials needed for a post-disaster emergency in the government material stockpile, to meet the demand for emergency materials in the early stage of the emergency. The production enterprise reserve means that the government assigns the task of stockpiling materials to enterprises, to give full play to the advantages of enterprise stockpiling.

Option contract theory is an important theory used in this study to address the coordination of government-enterprise cooperation interests. In drug stockpile management, a double marginal utility is caused by the inconsistency between the maximization of the respective interests pursued by the government and enterprises in the coordination of interests in the supply chain and the goal of maximizing the interests of collation [33, 34]. Options contracts, as an incentive mechanism for aligning supply chain interests, are frequently used in supply chain management. Under the option contract mechanism, a contract is drawn up between the two parties to the transaction whereby the ordering party commits itself to purchase a certain quantity of product in future periods, while it also purchases an option from the supplier [37]. Such options allow the ordering party to purchase a certain quantity of product in the future at a specified price, thereby gaining the right to adjust the number of future orders, ensure reliable supply, maintain stable prices, and meet uncertain demand.

In summary, the above two theories have laid an important foundation for the research of this paper, and have important guiding significance for the government-enterprise cooperative stockpile of drugs in shortage.

## 3 Problem description and assumptions

### 3.1 Problem description

Consider a supply chain of drugs in shortage consisting of the government and pharmaceutical enterprises, where the government and enterprises carry out joint stockpiling of drugs by signing an option cooperation agreement, with the emergence of a drug shortage as the trigger condition for the option. Government procurement consists of two parts: conventional procurement and flexible procurement. Conventional procurement refers to physical drugs (finished drugs) procured by the government through the signing of an option contract with an enterprise, with an upfront payment of royalties, by the parameters of the contract, as well as the environment of the region to which it belongs, demographic characteristics, and financial inventory constraints, among other factors. Flexible procurement means that the government incentivizes enterprises to stockpile a certain amount of production capacity and APIs for them through the agreed option exercise price, to make up for the demand for medicines for which their regular stockpiles are insufficient. Since most of the drugs in shortage are cheap essential drugs and the demand is uncertain, rational pharmaceutical enterprises often need to decide whether to participate in government cooperation based on their cost-benefit. Moreover, according to the type of enterprise production license division, pharmaceutical enterprises are usually divided into two categories: the formulation and API independent production enterprises, and API-F integrated production enterprises. If enterprises participate in this option cooperation, for the first group of enterprises, the formulation manufacturer will need to determine the amount of production capacity to be stockpiled based on the contractual parameters and conventional purchases provided by the government, and the API enterprise will need to determine the amount of API to be stockpiled based on the purchases and stockpiling by the government and the formulation manufacturer, as well as the government’s contractual parameters and the subsidy for the storage of the APIs. This is a significant case of decentralized decision-making in a three-tiered supply chain consisting of the government, the formulation enterprise, and the API enterprise. In the case of the API-F integrator, it needs to determine its production capacity stockpiles and API stockpiles based on the government’s conventional purchasing volume and contractual parameters, as well as the level of subsidy. This is a centralized decision-making scenario for a two-tier supply chain consisting of the government and the API-F manufacturer.

During the contract period, if there is a drug shortage, the government will first call on its conventional stockpile of drugs. When the conventional stockpiles are insufficient, flexible procurement will be initiated, whereby the enterprise will, in turn, urgently process the pre-stocked production capacity and APIs into finished products for the government’s needs. If the total amount of government and enterprise reserves still fails to meet the demand for drugs, the government will have to bear the corresponding shortage costs. If there is no drug shortage during the compact period, or if there is a surplus of materials at the end of the compact period, they will be handled by the salvage value.

### 3.2 Assumptions

To ensure the implementability of the model and with reference to some of the literature practices, the following assumptions are made in this paper:

1. The government-enterprise agreement is single-cycle, the contract length is equal to the shelf life of drugs, and when the reserve materials exceed the shelf life will be residual value treatment [32, 33, 54];
2. The agreement enterprise pre-storage production capacity and API does not exceed the government option order quantity, that is, the enterprise has no incentive to overstock [35, 70–72];
3. The government and the agreement enterprises are perfectly rational and have symmetric information [72–75];
4. Government subsidies, as well as enterprises’ capacity, are capped, and the government will bear the cost of shortages when demand is greater than the capacity range of the agreement firms [77–80].

### 3.3 Symbol description

The variable symbols and symbol meanings involved in this paper are shown in Table 1.

**Table 1:** Variable symbols and description.

Variable symbols	Symbol description
*x*	The random demand for a shortage-prone drug has a probability distribution function of *F*(*x*) and a maximum value of *U*.
*ρ*	Probability of a drug shortage.
*Q*	Conventional government stockpiles of drugs in shortage, i.e., physical stockpiles.
*q*_1_	Pharmaceutical production capacity stockpiles of the formulation.
*q*_2_	API suppliers’ API stockpiles.
*c*_1_	Production costs for conversion from an API to a finished drug product.
*c*_2_	Unit API production costs.
*c*_0_	Costs of urgent conversion from the production capacity to the finished drug.
*h*	Unit cost of stockpiling drug.
*m*_1_	Pharmaceutical enterprise’s pre-stocking production capacity costs, including the purchase of APIs after processing, pharmaceutical excipients, personnel and equipment preparation, and other costs.
*m*_2_	API suppliers pre-stock API costs, i.e., API stockpiling and management costs.
*p*	Unit initial purchase price of a finished drug.
*ω*	Unit API purchase price.
*υ*	Unit residual value of the drug in the form of physical stockpiles.
*r*_1_	Unit residual value of production capacity at the end of the period in the form of production capacity stockpiles.
*r*_2_	Unit residual value of API at the end of the period in the form of API stockpiles.
*o*	Unit option costs, i.e., agreed-upon premium
*e*	Unit option exercise price for the drug.
*s*	Unit API substitute storage subsidy is given to API suppliers when the government executes option purchases.
*M*	Unit drug stock-out costs.

### 3.4 Parameter constraints

Combined with the stockpile reality, there are the following parameter constraints:

1. *M*>*e*>*p*+*h*: The option exercise price needs to be greater than the sum of the initial purchase cost and stockpile cost of the drug, otherwise the enterprise will not participate in the cooperation; at the same time, the option exercise price needs to be less than the shortage cost, otherwise the government does not need to carry out flexible procurement.
2. *p*>*c*_1_+*c*_2_>*r*_1_+*r*_2_: The initial purchase price of the drug must be greater than the cost of production and the residual value must be less than the cost of production, otherwise the enterprise refuses to provide the drug.
3. *e*>*c*_0_+*m*_1_+*h*: The sum of the emergency processing of production capacity and the cost of stockpiling must be greater than the cost of stockpiling physical drugs and must be less than the agreed purchase price for an enterprise to stockpile production capacity.
4. *ω*>*s*>*m*_2_: The government’s subsidy to enterprises for the storage of APIs needs to be greater than the cost of the reserve of APIs, and at the same time less than the selling price of the APIs, otherwise the government and enterprises will not be able to reach an agreement on the stockpiling of APIs.

## 4 The benchmark model

### 4.1 Government separate stockpile model

Based on the elements considered in this study, and some of the references modeling process, we constructed the model for this study [32, 33, 83, 84]. The government’s separate stockpile of drugs in shortage is a government-managed stockpile, which means that before a drug shortage occurs, the government purchases a determined amount of drugs from pharmaceutical enterprises as a conventional stockpile at an initial purchase price, by the population of the region, historical monitoring data on drugs in shortage, and financial factors (*Q*_*a*_). If there is a drug shortage, the government utilizes this portion of the stockpile to meet the unexpected demand. Therefore, under the government’s separate stockpile model, the government’s cost function is shown in Eq (1):

$$\Pi_{g}^{a}=(p+h)Q_{a}-(1-\rho_{a})\upsilon Q_{a}+\rho_{a}(\overset{Q_{a}}{\underset{0}{\int }}-\upsilon (Q_{a}-x)f(x)dx+\overset{U}{\underset{Q_{a}}{\int }}M(x-Q_{a})f(x)dx)$$ (1)

In this equation, the first term is the cost of purchasing and stockpiling, the second term is the salvage benefit when there is no drug shortage, and the third term is the combination of salvage benefit and shortage cost for different demand states.

By solving the second-order condition for $\Pi_{g}^{a}$ concerning *Q*_*a*_, we obtain: $\frac{\partial^{2}\prod_{a}^{g}}{\partial Q_{a}^{2}}=\rho_{a}(M-\upsilon )f(Q_{a})>0$, which shows that there exists an optimal reserves $Q_{a}^{*}$ that minimizes the cost to the government, as shown in Eq (2):

$$Q_{a}^{*}=F^{-1}(1-\frac{p+h-\upsilon }{\rho_{a}(M-\upsilon )})$$ (2)

Therefore, the optimal stockpile cost ($\Pi_{g}^{a^{*}}$) for the government under the government’s separate stockpile model is as follows:

$$\Pi_{g}^{a^{*}}=Q_{a}^{*}(p+h-v)+\rho_{a}[\upsilon (Q_{a}^{*}-\int_{0}^{a_{a}^{*}}F(x)d(x))+M(U-Q_{a}^{*}-\int_{Q_{a}^{*}}^{U}F(x)d(x))]$$ (3)

As previously mentioned, there are two types of government purchasing channels, which respectively constitute a decentralized decision-making situation for a three-tier supply chain consisting of the government, the formulation manufacturer, and the API enterprise, and a centralized decision-making situation for a two-tier supply chain consisting of the government and the API-F integrated enterprise. Regardless of which channel, the enterprises’ optimal profit stems from the government’s explicit purchase quantity. For decentralized decision-making firms, the optimal profit of the formulation manufacturer ($\Pi_{m}^{a^{*}}$) and the optimal profit of the API supplier ($\Pi_{s}^{a^{*}}$) are respectively:

$$\Pi_{m}^{a^{*}}=Q_{a}^{*}(p-c_{1}-\omega )$$ (4)

$$\prod_{s}^{a^{*}}=Q_{a}^{*}(\omega -c_{2})$$ (5)

For an API-F integrated enterprise, the profit of the enterprise is:

$$\Pi_{e}^{a^{*}}=Q_{a}^{*}(p-c_{1}-c_{2})$$ (6)

### 4.2 Supply chain centralized decision-making model

The centralized decision-making model of the supply chain is one in which the government and pharmaceutical enterprises as a whole make decisions together. In this case, the government is the centralized decision maker, and together with the pharmaceutical enterprises, they plan to produce a certain amount of drugs as the system reserves (*Q*_*s*_). Therefore, the system cost function under the supply chain centralized decision-making model is shown in Eq (7):

$$\Pi_{sc}^{a}=(c_{1}+c_{2}+h)Q_{s}-\upsilon (1-\rho )Q_{s}+\rho_{a}[-\int_{0}^{Q_{s}}\upsilon (Q_{s}-x)f(x)dx+\int_{Q_{s}}^{U}M(x-Q_{s})f(x)dx]$$ (7)

In this equation, the first term is the production and stockpiling costs, the second term is the residual benefit when there are no drug shortages, and the third term is the combination of residual benefit and shortage cost for different demand states.

By solving the second-order condition of $\Pi_{sc}^{a}$ concerning *Q*_*s*_, we obtain: $\frac{\partial^{2}\prod_{sc}^{a}}{\partial Q_{s}^{2}}=\rho_{s}(M-\upsilon )f(Q_{s})>0$, which shows that there exists an optimal stockpile $Q_{s}^{*}$ that minimizes the cost to the government, as shown in Eq (8):

$$Q_{s}^{*}=F^{-1}(1-\frac{c_{1}+c_{2}+h-\upsilon }{\rho_{s}(M-\upsilon )})$$ (8)

Therefore, the system cost under centralized decision making in the supply chain is:

$$\Pi_{sc}^{a^{*}}=Q_{s}^{*}\left(c_{1}+c_{2}+h-v\right)+\rho_{s}[\upsilon (Q_{s}^{*}-\int_{0}^{Q_{s}^{*}}F(x)d(x))+M(U-Q_{s}^{*}-\int_{Q_{s}^{*}}^{U}F(x)d(x))]$$ (9)

## 5 Model construction and solution

### 5.1 Decentralized decision model

In the decentralized decision-making situation, the government establishes a cooperative relationship with the formulation manufacturers and the API supplier through option contracts and incentivizes enterprises to stockpile a certain amount of materials for them through flexible procurement prices and subsidies for the storage of APIs. The enterprises, to maximize their own expected returns, decide whether to participate in the agreement to stockpile, as well as the amount of stockpile. According to the description of this problem, the decision-making sequence of the government, the formulation manufacturer, and the API supplier is as follows:

1. In the process of prior stockpiling in the event of a drug shortage, the government, through the release of bidding information on behalf of the stockpile and the enterprise agreement on the parameters of the option contract, provides the participating enterprises with a unified option premium *o* per unit and reaches an agreement with the enterprise on the option exercise price *e* of the drug and subsidies *s* of the stockpile of APIs, to incentivize the pharmaceutical enterprises to stockpile a certain amount of production capacity and APIs for itself.
2. Based on the parameters of the contract, as well as the demographic, financial, and environmental factors of the region in which it operates, the government purchases a certain quantity *Q*_*b*_ of drugs from the formulation manufacturer at a price per unit *p* as its conventional reserves.
3. The formulation manufacturer determines the production capacity reserves $q_{1}^{b}$ based on the option price parameters provided by the government; the API manufacturer determines the API reserves $q_{2}^{b}$ based on the production capacity reserves of the formulation manufacturer and the government subsidy level.
4. Whether there is a drug shortage or not, the fixed cost to the government is $Q_{b}(p+h)+o(q_{1}^{b}+q_{2}^{b})$, the fixed profit to the formulation manufacturer is $Q_{b}(p-\omega -c_{1})+oq_{1}^{b}-q_{1}^{b}(m_{1}+\omega )$, and the fixed profit to the API supplier is $\left(Q_{b}+q_{1}^{b})(\omega -c_{2})+oq_{2}^{b}-q_{2}^{b}(m_{2}+c_{2}\right)$.
5. If there is no shortage of drugs during the reserve cycle (with probability 1−*ρ*_*b*_), the government does not need to carry out flexible procurement of drugs, and the stockpile materials of the government and enterprises are all treated according to the salvage value, with the salvage value gain of the government as *νQ*_*b*_, the salvage value gain of the formulation manufacturer as $r_{1}q_{1}^{b}$, and the salvage value gain of the API supplier as $r_{2}q_{2}^{b}$.
6. If there is a drug shortage (with probability *ρ*_*b*_), the costs or profits of both the government and the pharmaceutical enterprise vary with the demand for drug *x*, as follows:

➀ When 0<*x*≤*Q*_*b*_, the government does not need to engage in flexible procurement because its conventional reserves are sufficient to meet the demand for drugs. In this case, the government’s gain is the residual value gain *ν*(*Q*_*b*_−*x*) of part of the conventional reserves of drugs, the residual value gain $r_{1}q_{1}^{b}$ of the formulation manufacturer, and the residual value gain $r_{2}q_{2}^{b}$ of the API supplier.
➁ When $Q_{b}<x\leq Q_{b}+q_{1}^{b}$, the government’s conventional reserves cannot meet the demand for drugs, and it is necessary for the formulation manufacturer to convert part of its pre-stockpile production capacity into a supply of finished drugs. At this time, the cost of the government is *e*(*x*−*Q*_*b*_), the formulation manufacturer’s revenue is $\left(e-c_{0})(x-Q_{b})+r_{1}(Q_{b}+q_{1}^{b}-x\right)$, and the API supplier’s revenue is the salvage value revenue $r_{2}q_{2}^{b}$.
➂ When $Q_{b}+q_{1}^{b}<x\leq Q_{b}+q_{1}^{b}+q_{2}^{b}$, the production capacity of the formulation manufacturer is also unable to meet the demand for drugs, and it is necessary to purchase further APIs from the API supplier for emergency processing to produce finished drugs. At this time, the cost to the government is $e(x-Q_{b})+s(x-Q_{b}-q_{1}^{b})$, the formulation manufacturer’s revenue is $\left(e-c_{0})q_{1}^{b}+(e-c_{1}-\omega )(x-Q_{b}-q_{1}^{b}\right)$, and the API supplier’s revenue is $\omega (x-Q_{b}-q_{1}^{b})+r_{2}(Q_{b}+q_{1}^{b}+q_{2}^{b}-x)+s(x-Q_{b}-q_{1}^{b})$.
➃ When $Q_{b}+q_{1}^{b}+q_{2}^{b}<x\leq U$, since both the government and the enterprises stockpile supplies to meet the demand for drugs, at this time, the government, in addition to paying the cost of option exercise of enterprises, also needs to bear the cost of shortage of goods, i.e., $e(q_{1}^{b}+q_{2}^{b})+M(x-Q_{b}-q_{1}^{b}-q_{2}^{b})+sq_{2}^{b}$, the formulation manufacturer’s revenue is $(e-c_{0})q_{1}^{b}+(e-c_{1}-\omega )q_{2}^{b}$, and the API supplier’s revenue is $\omega q_{2}+sq_{2}^{b}$.

From the above decision sequence, it can be seen that this is a Stackelberg game with the government as the dominant player and the enterprises as the subordinates. Each party makes decisions to maximize its benefits, in which the decision variable of the government is the drug conventional reserves *Q*_*b*_, the decision variable of the formulation manufacturer is the production capacity reserves $q_{1}^{b}$, and the decision variable of the API enterprise is the API reserves $q_{2}^{b}$. Therefore, the next model analysis will be to derive the optimal decision of each party through the backward derivation-solving method.

#### 5.1.1 Decision analysis of the API supplier

After the above analysis, the profit function of the API supplier is:

$$\begin{array}{l} \Pi_{s}^{b}=[\left(Q_{b}+q_{1}^{b})(\omega -c_{2})+oq_{2}^{b}-q_{2}^{b}(m_{2}+c_{2}\right)]+(1-\rho )r_{2}q_{2}^{b}+\rho [\int_{0}^{Q_{b}+q_{1}^{b}}r_{2}q_{2}^{b}f(x)dx\\ \;+\int_{Q_{b}+q_{1}^{b}}^{Q_{b}+q_{1}^{b}+q_{2}^{b}}(\omega (x-Q_{b}-q_{1}^{b})+r_{2}(Q_{b}+q_{1}^{b}+q_{2}^{b}-x)+s(x-Q_{b}-q_{1}^{b}))f(x)dx\\ \;+\int_{Q_{b}+q_{1}^{b}+q_{2}^{b}}^{U}(\omega q_{2}^{b}+sq_{2}^{b})f(x)dx] \end{array}$$ (10)

The first item is the fixed revenue of the API supplier, the second item is the residual revenue when there is no drug shortage, and the third item is a combination of the residual revenue and the revenue from the API sold under different demand states.

Solving Eq (10) for the first and second order in verses concerning the API reserves *q*_2_ yields:

$$\frac{\partial \prod_{s}^{b}}{\partial q_{2}^{b}}=(o-m_{2}-c_{2}+r_{2})+\rho [\left(r_{2}-\omega -s)(F(Q_{b}+q_{1}^{b}+q_{2}^{b})-1\right)]$$ (11)

$$\frac{\partial^{2}\Pi_{s}^{b}}{\partial^{2}q_{2}^{b}}=\rho \left(r_{2}-\omega -s\right)f\left(Q_{b}+q_{1}^{b}+q_{2}^{b}\right)<0$$ (12)

According to the parametric conditions, it is obtained that $\frac{\partial \prod_{s}^{b}}{\partial q_{2}^{b}}<0$ is constant, indicating that there exists an optimal API reserves $q_{2}^{b^{*}}$ that maximizes the profit of the API supplier:

$$q_{2}^{b^{*}}=F^{-1}[1-\frac{o-m_{2}-c_{2}+r_{2}}{\rho (r_{2}-\omega -s)}]-Q_{b}-q_{1}^{b}$$ (13)

According to the expression of the optimal reserves of the API, it can be seen that it is not only related to the production cost of the API, the cost of pre-stored API, the sold price of the API, the government subsidy and other factors, but also related to the decision-making of the government and the formulation manufacturer. Therefore, the decisions of these two are further solved by inverse order derivation.

#### 5.1.2 Decision analysis of the formulation manufacturer

Under the decentralized decision model, the profit function of the formulation manufacturer is:

$$\begin{array}{l} \Pi_{m}^{b}=[Q_{b}(p-\omega -c_{1})+oq_{1}^{b}-q_{1}^{b}(m_{1}+\omega )]+(1-\rho )r_{1}q_{1}\\ \;+\rho [\int_{0}^{Q_{b}}r_{1}q_{1}^{b}f(x)dx+\int_{Q_{b}}^{Q_{b}+q_{1}^{b}}(\left(e-c_{0})(x-Q_{b})+r_{1}(Q_{b}+q_{1}^{b}-x\right))f(x)dx\\ \;+\int_{Q_{b}+q_{1}^{b}}^{Q_{b}+q_{1}^{b}+q_{2}^{b}}(\left(e-c_{0})q_{1}^{b}+(e-c_{1}-\omega )(x-Q_{b}-q_{1}^{b}\right))f(x)dx\\ \;+\int_{Q_{b}+q_{1}^{b}+q_{2}^{b}}^{U}((e-c_{0})q_{1}^{b}+(e-c_{1}-\omega )q_{2}^{b})f(x)dx] \end{array}$$ (14)

The first item is the fixed profit of the formulation manufacturer, the second item is the residual value gain when there is no drug shortage, and the third item is the combination of the residual value gain and the drug processing cost and sale proceeds under different demand states.

Similarly, solving for the first and second-order inverses of Eq (14) concerning the production capacity reserves $q_{1}^{b}$ yields:

$$\frac{\partial \prod_{m}^{b}}{\partial q_{1}^{b}}=(o-m_{1}-\omega +r_{1})+\rho [\left(r_{1}+c_{0}-c_{1}-\omega )(F(Q_{b}+q_{1}^{b})-1)-(e-c_{1}-\omega )(F(Q_{b}+q_{1}^{b}+q_{2}^{b})-1\right)]$$ (15)

$$\frac{\partial^{2}\Pi_{m}^{b}}{\partial^{2}q_{1}^{b}}=\rho [\left(r_{1}+c_{0}-c_{1}-\omega )f(Q_{b}+q_{1}^{b})-(e-c_{1}-\omega )f(Q_{b}+q_{1}^{b}+q_{2}^{b}\right)]<0$$ (16)

According to the parametric conditions, it is obtained that $\frac{\partial \prod_{m}^{b}}{\partial q_{1}^{b}}<0$ is constant, indicating that there exists an optimal production capacity reserves that maximize the profitability of the formulation manufacturer, $q_{1}^{b^{*}}$. This is obtained by solving (15) and Eq (11):

$$q_{1}^{b^{*}}=F^{-1}[1-\frac{\left(c_{1}+\omega -e)(o-m_{2}-c_{2}+r_{2})+(o-m_{1}-\omega +r_{1})(\omega +s-r_{2}\right)}{\rho (\omega +s-r_{2})(r_{1}+c_{0}-c_{1}-\omega )}]-Q_{b}$$ (17)

#### 5.1.3 Decision analysis of the government

Under the decentralized decision-making model, the government’s cost function is:

$$\begin{array}{l} \Pi_{g}^{b}=[Q_{b}(p+h)+o(q_{1}^{b}+q_{2}^{b})]-(1-\rho )\upsilon Q_{b}+\rho [\int_{0}^{Q_{b}}-\nu (Q_{b}-x)f(x)dx\\ \;+\int_{Q_{b}}^{Q_{b}+q_{1}^{b}}e(x-Q_{b})f(x)dx+\int_{Q_{b}+q_{1}^{b}}^{Q_{b}+q_{1}^{b}+q_{2}^{b}}(e(x-Q_{b})+s(x-Q_{b}-q_{1}^{b}))f(x)dx\\ \;+\int_{Q_{b}+q_{1}^{b}+q_{2}^{b}}^{U}(e(q_{1}^{b}+q_{2}^{b})+M(x-Q_{b}-q_{1}^{b}-q_{2}^{b})+sq_{2}^{b})f(x)dx] \end{array}$$ (18)

In this equation, the first item is the government’s fixed cost, which contains the cost of purchasing and stockpiling conventional stockpiles, option premiums, and subsidies for substitute stockpiles, the second item is the residual return on conventional stockpiles when there is no drug shortage, and the third item is a combination of residual returns on stockpiled drugs, flexible purchasing costs, and shortage costs in the context of varying levels of demand for medicines.

When the supply chain reaches the coordinated state, there exists $Q_{b}+q_{1}^{*}+q_{2}^{*}=Q_{s}^{*}$. Therefore, $q_{1}^{*}+q_{2}^{*}$ in Eq (19) can be expressed as $Q_{s}^{*}-Q_{b}$. Further solving the first and second-order derivatives of Eq (18) concerning *Q*_*b*_ yields:

$$\frac{\partial \Pi_{g}^{b}}{\partial Q_{b}}=(p+h-o-\upsilon )+\rho [\left(e-\upsilon )(F(Q_{b})-1)-s(F(Q_{b}+q_{1}^{b}+q_{2}^{b})-F(Q_{b}+q_{1}^{b})\right)]$$ (19)

$$\frac{\partial^{2}\Pi_{g}^{b}}{\partial^{2}Q_{b}}=\rho [\left(e-\upsilon )f(Q_{b})-s(f(Q_{b}+q_{1}^{b}+q_{2}^{b})-f(Q_{b}+q_{1}^{b})\right)]>0$$ (20)

Since $\frac{\partial^{2}\Pi_{g}^{b}}{\partial^{2}Q_{b}}>0$ is constant, it shows that there exists optimal reserves $Q_{b}^{*}$ that minimize the cost to the government under this model, and then associating Eqs (19), (11), and (15) yields $Q_{b}^{*}$, as shown in Eq (21).


$$Q_{b}^{*}=F^{-1}(1-\frac{(p+h-o-\upsilon )-sA}{\rho (e-\upsilon )})$$ (21)

where $A=\frac{\left(o-m_{2}-c_{2}+r_{2})(e-c_{0}-r_{1})+(o-m_{1}-\omega +r_{1})(r_{2}-\omega -s\right)}{\left(r_{2}-\omega -s)(r_{1}+c_{0}-c_{1}-\omega \right)}$.

#### 5.1.4 Supply chain coordination analysis

If the coordination of the supply chain system of drugs in shortage is to be realized, it is necessary to satisfy $Q_{b}^{*}+q_{1}^{b^{*}}+q_{2}^{b^{*}}=Q_{s}^{*}$. Referring to the complexity of the model established in this paper, if the specific form of the distribution of drug demand in the event of a drug shortage is unknown, it will be even more impossible to analyze the equilibrium decision-making of the government-enterprise tripartite, as well as their respective costs and profits. Therefore, referring to the practice of literature [58, 60, 63], it is assumed that the demand obeys a uniform distribution, i.e., $F(x)=\frac{x}{U}$.

**Proposition 1** In the decentralized decision-making situation of government-enterprise option cooperation, the optimal physical reserves of drugs in shortage for the government, the optimal production capacity reserves for the formulation manufacturer, and the optimal API reserves for the API supplier are:

$$\{\begin{array}{l} Q_{b}^{*}=F^{-1}(1-\frac{(p+h-o-\upsilon )-sA}{\rho (e-\upsilon )})\\ q_{1}^{b_{1}^{*}}=F^{-1}[1-\frac{\left(c_{1}+\omega -e)(o-m_{2}-c_{2}+r_{2})+(o-m_{1}-\omega +r_{1})(\omega +s-r_{2}\right)}{\rho (\omega +s-r_{2})(r_{1}+c_{0}-c_{1}-\omega )}]-Q_{b}^{*}\\ q_{2}^{b^{*}}=F^{-1}[1-\frac{o-m_{2}-c_{2}+r_{2}}{\rho (r_{2}-\omega -s)}]-Q_{b}^{*}-q_{1}^{b*} \end{array}$$ (22)

Proposition 1 expresses the optimal stockpiling decision of each party in the decentralized decision-making situation of government-enterprise option cooperation. To ensure the validity of the model established in this section, it is necessary to ensure that the three decision variables in Eq (22) are all greater than 0, according to which the constraints on the probability of drug shortages, the option exercise price, and the government subsidy are obtained: enterprises choose to participate in the government-enterprise stockpiling cooperation for drugs in shortage when *ρ* satisfies $\rho >\frac{(p+h-o-\upsilon )-sA}{e-\upsilon }$, when *e* satisfies $\frac{\left(o-m_{1}-\omega +r_{1})(\omega +s-r_{2}\right)}{o-m_{2}-c_{2}+r_{2}}+c_{0}+r_{1}<e<M$, and when *s* satisfies $s>\frac{\left(M-\upsilon )(m_{2}+c_{2}-o-r_{2}\right)}{c_{1}+c_{2}+h-\upsilon }+r_{2}-\omega$. Otherwise, the enterprises’ expected returns cannot make up for the losses brought by their risk-taking.

**Proposition 2** In the decentralized decision-making situation of government-enterprise option cooperation, the supply chain of drugs in shortage reaches a coordinated state when $s=\frac{\left(M-\upsilon )(m_{2}+c_{2}-o-r_{2}\right)}{c_{1}+c_{2}+h-\upsilon }+r_{2}-\omega$.

Proposition 2 gives a condition for the supply chain to reach a coordinated state in the decentralized decision-making situation. It can be found that this coordination condition is only related to the substitute stockpile subsidy *s*, but not to the option exercise price *e*. Further, solving the first-order conditions on the shortage cost *M*, the API production cost *c*_2_, and the API stockpile cost *m*_2_, we obtain $\frac{\partial s}{\partial M}>0$, $\frac{\partial s}{\partial c_{2}}>0$, and $\frac{\partial s}{\partial m_{2}}>0$, which show that the government’s substitute stockpile subsidy increases with the increase of the shortage cost, the API production cost, and the stockpile cost.

**Proposition 3** A first-order derivation of the optimal stockpile for the government and enterprises concerning the probability of drug shortage *ρ*, the option exercise price *e*, and the government substitute stockpile subsidy *s* yields: $\frac{\partial Q_{b}^{*}}{\partial \rho }>0$, $\frac{\partial q_{1}^{b^{*}}}{\partial \rho }>0$, $\frac{\partial q_{2}^{b^{*}}}{\partial \rho }<0$; $\frac{\partial Q_{b}^{*}}{\partial e}>0$, $\frac{\partial q_{1}^{b^{*}}}{\partial e}<0$, $\frac{\partial q_{2}^{b^{*}}}{\partial e}>0$; $\frac{\partial Q_{b}^{*}}{\partial s}<0$, $\frac{\partial q_{1}^{b^{*}}}{\partial s}<0$, $\frac{\partial q_{2}^{b^{*}}}{\partial s}>0$.

Proposition 3 shows that in the decentralized decision-making situation, the government’s optimal conventional reserves $Q_{b}^{*}$ increase with the probability of drug shortage *ρ* and the option exercise price *e*, and decrease with the government’s substitute stockpile subsidy *s*. The optimal production capacity reserves $q_{1}^{b^{*}}$ of the formulation manufacturer increase with the probability of drug shortage *ρ* and decrease with the option exercise price *e* and the substitute stockpile subsidy *s*. The optimal API reserves $q_{1}^{b^{*}}$ of the API supplier decrease with the probability of drug shortage *ρ*, and increase with the option exercise price *e* and the government’s substitute stockpile subsidy *s*.

**Proposition 4** Based on the above results, it can be obtained that in the decentralized decision-making situation of government-enterprise option cooperation, the costs and profits of government-enterprise tripartite decision-making to reach the equilibrium state are respectively:

$$\begin{array}{l} \Pi_{g}^{b^{*}}=[Q_{b}^{*}(p+h)+o(q_{1}^{b^{*}}+q_{2}^{b^{*}})]-(1-\rho )\upsilon Q_{b}^{*}+\rho [\int_{0}^{Q_{b}^{*}}-\nu (Q_{b}^{*}-x)f(x)dx\\ \;+\int_{Q_{b}^{*}}^{Q_{b}^{*}+q_{1}^{b^{*}}}e(x-Q_{b}^{*})f(x)dx+\int_{Q_{b}^{*}+q_{1}^{b^{*}}}^{Q_{b}^{*}+q_{1}^{b^{*}}+q_{2}^{b^{*}}}\left(e(x-Q_{b}^{*})+s(x-Q_{b}^{*}-q_{1}^{b^{*}})\right)f(x)dx\\ \;+\int_{Q_{b}^{*}+q_{1}^{b^{*}}+q_{2}^{b^{*}}}^{U}\left(e(q_{1}^{b^{*}}+q_{2}^{b^{*}})+M(x-Q_{b}^{*}-q_{1}^{b^{*}}-q_{2}^{b^{*}})+sq_{2}^{b^{*}}\right)f(x)dx] \end{array}$$ (23)

$$\begin{array}{l} \Pi_{m}^{b^{*}}=[Q_{b}^{*}(p-\omega -c_{1})+oq_{1}^{b^{*}}-q_{1}^{b^{*}}(m_{1}+\omega )]+(1-\rho )r_{1}q_{1}^{b^{*}}\\ \;+\rho [\int_{0}^{Q_{b}^{*}}r_{1}q_{1}^{b^{*}}f(x)dx+\int_{Q_{b}^{*}}^{Q_{b}^{*}+q_{1}^{b^{*}}}\left(\left(e-c_{0})(x-Q_{b}^{*})+r_{1}(Q_{b}^{*}+q_{1}^{b^{*}}-x\right)\right)f(x)dx\\ \;+\int_{Q_{b}^{*}+q_{1}^{b^{*}}}^{Q_{b}^{*}q_{1}^{b^{*}}+q_{2}^{b^{*}}}\left(\left(e-c_{0})q_{1}^{b^{*}}+(e-c_{1}-\omega )(x-Q_{b}^{*}-q_{1}^{b^{*}}\right)\right)f(x)dx\\ \;+\int_{Q_{b}^{*}+q_{1}^{b^{*}}+q_{2}^{b^{*}}}^{U}\left((e-c_{0})q_{1}^{b^{*}}+(e-c_{1}-\omega )q_{2}^{b^{*}}\right)f(x)dx] \end{array}$$ (24)

$$\begin{array}{l} \Pi_{s}^{b^{*}}=[\left(Q_{b}^{*}+q_{1}^{b^{*}})(\omega -c_{2})+oq_{2}^{b^{*}}-q_{2}^{b^{*}}(m_{2}+c_{2}\right)]+(1-\rho )r_{2}q_{2}^{b^{*}}+\rho [\int_{0}^{Q_{b}^{*}+q_{1}^{b^{*}}}r_{2}q_{2}^{b^{*}}f(x)dx\\ \;+\int_{Q_{b}^{*}+q_{1}^{b^{*}}}^{Q_{b}^{*}+q_{1}^{b^{*}}+q_{2}^{b^{*}}}\left(\omega (x-Q_{b}^{*}-q_{1}^{b^{*}})+r_{2}(Q_{b}^{*}+q_{1}^{b^{*}}+q_{2}^{b^{*}}-x)+s(x-Q_{b}^{*}-q_{1}^{b^{*}})\right)f(x)dx\\ \;+\int_{Q_{b}^{*}+q_{1}^{b^{*}}+q_{2}^{b^{*}}}^{U}\left(\omega q_{2}^{b^{*}}+sq_{2}^{b^{*}}\right)f(x)dx] \end{array}$$ (25)

### 5.2 Centralized decision model

The centralized decision-making model developed in this section applies to the API-F integrated enterprise. The government first gives the option contract parameters and the substitute stockpiling subsidy amount, and the enterprise decides its production capacity reserves and API reserves with the goal of profit maximization. The specific decision sequence is as follows:

1. During the ex-ante stockpiling process in the event of a drug shortage, the government incentivizes the enterprise to stockpile a certain amount of production capacity and APIs by releasing information on the bidding for stockpiling on behalf of the enterprise and agreeing on the parameters of the option contract, including the option premium *o* and the option exercise price *e* and the API stockpiling subsidy *s* for each unit of the drug.
2. Based on the parameters of the contract and the demographic, financial, and environmental factors of the region in which it operates, the government purchases a certain quantity of drugs from the firms at a price per unit *p* as its conventional stockpile quantity *Q*_*c*_.
3. The enterprise determines the production capacity reserves $q_{1}^{c}$ and API reserves $q_{2}^{c}$ based on the contractual parameters provided by the government.
4. Regardless of whether or not there is a drug shortage, the fixed cost to the government is $Q_{c}(p+h)+o(q_{1}^{c}+q_{2}^{c})$ and the fixed revenue to the enterprise is $Q_{c}(p-c_{1}-c_{2})-q_{1}^{c}(c_{2}+m_{1})-q_{2}^{c}(c_{2}+m_{2})+o(q_{1}^{c}+q_{2}^{c})$.
5. If there is no drug shortage during the stockpile cycle (with probability 1−*ρ*), then the government does not need to carry out flexible procurement of drugs, and the government and the enterprise stockpile materials are handled by the salvage value upon expiration, with the government’s salvage value gain *νQ*_*c*_, and the enterprise’s salvage value gain $r_{1}q_{1}^{c}+r_{2}q_{2}^{c}$.
6. If there is a drug shortage (with probability *ρ*), the costs or profits of both the government and the pharmaceutical enterprise vary with the demand for drug *x*, as follows:

When 0<*x*≤*Q*_*c*_, the government’s conventional reserves are sufficient to meet the demand for drugs, and there is no need for flexible procurement. At this time, the government’s gain is the residual value gain *ν*(*Q*_*c*_−*x*) of some of the conventional stockpiled drugs, and the residual value gain of the enterprise’s stockpiled materials is $r_{1}q_{1}^{c}+r_{2}q_{2}^{c}$.

When $Q_{c}<x\leq Q_{c}+q_{1}^{c}$, the government’s conventional reserves can not meet the demand for drugs, but also need to carry out part of the production capacity of flexible procurement. At this time, the cost of the government is *e*(*x*−*Q*_*c*_), and the enterprise’s revenue is $(e-c_{0})(x-Q_{c})+r_{1}(Q_{c}+q_{1}^{c}-x)+r_{2}q_{2}^{c}$.

When $Q_{c}+q_{1}^{c}<x\leq Q_{c}+q_{1}^{c}+q_{2}^{c}$, the production capacity of the enterprise is unable to meet the demand for drugs, and further urgent processing of pre-stored APIs into finished drugs is required. The cost to the government at this time is $e(x-Q_{c})+s(x-Q_{c}-q_{1}^{c})$ and the revenue to the enterprise is $\left(e-c_{0})q_{1}^{c}+(e+s-c_{1})(x-Q_{c}-q_{1}^{c})+r_{2}(Q_{c}+q_{1}^{c}+q_{2}^{c}-x\right)$.

When $Q_{c}+q_{1}^{c}+q_{2}^{c}<x\leq U$, since both the government and the enterprise stockpile materials are unable to meet the demand for drugs, at this time, the government needs to pay the cost of the enterprise as well as to bear the cost of the shortage of drugs, i.e., $e(q_{1}^{c}+q_{2}^{c})+sq_{2}^{c}+M(x-Q_{c}-q_{1}^{c}-q_{2}^{c})$, and the enterprise’s revenue is $(e-c_{0})q_{1}^{c}+(e+s-c_{1})q_{2}^{c}$.

As well, this is a Stackelberg game with the government as the dominant and the enterprise as the subordinate. Next, the optimal stockpiling decisions of both the government and the enterprise are derived by the backward derivation solution method.

#### 5.2.1 Decision analysis of the enterprise

As a result of the above analysis, the profit equation of the API-F integrated enterprise is:

$$\begin{array}{l} \Pi_{e}^{c}=[Q_{c}(p-c_{1}-c_{2})-q_{1}^{c}(c_{2}+m_{1})-q_{2}^{c}(c_{2}+m_{2})+o(q_{1}^{c}+q_{2}^{c})]+(1-\rho )(r_{1}q_{1}^{c}+r_{2}q_{2}^{c})\\ \;+\rho \int_{0}^{Q_{c}}\left(r_{1}q_{1}^{c}+r_{2}q_{2}^{c}\right)f(x)dx+\int_{Q_{c}}^{Q_{c}+q_{1}^{c}}\left((e-c_{0})(x-Q_{c})+r_{1}(Q_{c}+q_{1}^{c}-x)+r_{2}q_{2}^{c}\right)f(x)dx\\ \;+\int_{Q_{c}+q_{1}^{c}}^{Q_{c}+q_{1}^{c}+q_{2}^{c}}\left(\left(e-c_{0})q_{1}^{c}+(e+s-c_{1})(x-Q_{c}-q_{1}^{c})+r_{2}(Q_{c}+q_{1}^{c}+q_{2}^{c}-x\right)\right)f(x)dx\\ \;+\int_{Q_{c}+q_{1}^{c}+q_{2}^{c}}^{U}\left((e-c_{0})q_{1}^{c}+(e+s-c_{1})q_{2}^{c}\right)f(x)dx] \end{array}$$ (26)

In this equation, the first item is the fixed profit of the enterprise, including the revenue from the sale of the regular reserve quantity, the cost of production capacity stockpile, the cost of API stockpile, premiums, and substitute stockpile subsidies, the second item is the residual value of the stockpiled drugs in case of no drug shortage, and the third item is the combination of the residual value of the stockpiled drugs and the revenue obtained from the government’s flexible purchasing in the background of the demand for different drugs.

Solving Eq (26) for the first-order derivative and second-order inverse, as well as partial derivatives, concerning the production capacity reserves $q_{1}^{C}$ and the API reserves $q_{2}^{C}$, yields:

$$\frac{\partial \prod_{e}^{c}}{\partial q_{1}^{c}}=(r_{1}+o-m_{1}-c_{2})+\rho [\left(r_{1}+c_{0}+s-c_{1}-r_{2})(F(Q_{c}+q_{1}^{c})-1)+(c_{1}+r_{2}-e-s)(F(Q_{c}+q_{1}^{c}+q_{2}^{c})-1\right)]$$ (27)

$$\frac{\partial^{2}\prod_{e}^{c}}{\partial^{2}q_{1}^{c}}=\rho [\left(r_{1}+c_{0}+s-c_{1}-r_{2})f(Q_{c}+q_{1}^{c})+(c_{1}+r_{2}-e-s)f(Q_{c}+q_{1}^{c}+q_{2}^{c}\right)]<0$$ (28)

$$\frac{\partial^{2}\Pi_{e}^{c}}{\partial q_{1}^{c}q_{2}^{c}}=\rho (c_{1}+r_{2}-e-s)f(Q_{c}+q_{1}^{c}+q_{2}^{c})$$ (29)

$$\frac{\partial \prod_{e}^{c}}{\partial q_{2}^{c}}=(r_{2}+o-m_{2}-c_{2})+\rho [\left(c_{1}+r_{2}-e-s)(F(Q_{c}+q_{1}^{c}+q_{2}^{c})-1\right)]$$ (30)

$$\frac{\partial^{2}\prod_{e}^{c}}{\partial^{2}q_{2}^{c}}=\rho (c_{1}+r_{2}-e-s)f(Q_{c}+q_{1}^{c}+q_{2}^{c})<0$$ (31)

$$\frac{\partial^{2}\Pi_{e}^{c}}{\partial q_{2}^{c}q_{1}^{c}}=\rho (c_{1}+r_{2}-e-s)f(Q_{c}+q_{1}^{c}+q_{2}^{c})$$ (32)

Solving the Hessian matrix yields: $H=\rho^{2}(r_{1}+c_{0}+s-c_{1}-r_{2})(c_{1}+r_{2}-e-s)\times f(Q_{c}+q_{1}^{c})f(Q_{c}+q_{1}^{c}+q_{2}^{c})>0$, indicating that under the centralized decision model, there exists the optimal production capacity reserves $q_{1}^{c^{*}}$ and the optimal API reserves $q_{2}^{c^{*}}$ to maximize the profit of the enterprise:

$$q_{1}^{c^{*}}=F^{-1}[1-\frac{r_{1}-r_{2}-m_{1}+m_{2}}{\rho (r_{1}+c_{0}+s-c_{1}-r_{2})}]-Q_{c}$$ (33)

$$q_{2}^{c^{*}}=F^{-1}[1-\frac{r_{2}+o-m_{2}-c_{2}}{\rho (c_{1}+r_{2}-e-s)}]-Q_{c}-q_{1}^{c}$$ (34)

#### 5.2.2 Decision analysis of the government

Under centralized decisions, the government’s cost function is:

$$\begin{array}{l} \Pi_{g}^{b}=[Q_{c}(p+h)+o(q_{1}^{c}+q_{2}^{c})]-(1-\rho )\upsilon Q_{c}+\rho [\int_{0}^{Q_{c}}-\nu (Q_{c}-x)f(x)dx\\ \;+\int_{Q_{c}}^{Q_{c}+q_{1}^{c}}e(x-Q_{c})f(x)dx+\int_{Q_{c}}^{Q_{c}+q_{1}^{c}+q_{2}^{c}}(e(x-Q_{c})+s(x-Q_{c}-q_{1}^{c}))f(x)dx\\ \;+\int_{Q_{c}+q_{1}^{c}+q_{2}^{c}}^{U}(e(q_{1}^{c}+q_{2}^{c})+sq_{2}^{c}+M(x-Q_{c}-q_{1}^{c}-q_{2}^{c}))f(x)dx] \end{array}$$ (35)

In this equation, the first item is the government’s fixed cost, the second item is the salvage value gain when there is no drug shortage, and the third item is a combination of the salvage value gain of stockpiled drugs, the cost of flexible purchasing, and the cost of shortages for different drug demand states.

When the supply chain reaches the coordinated state, there exists $Q_{c}+q_{1}^{c^{*}}+q_{2}^{c^{*}}=Q_{s}^{*}$. Therefore, $q_{1}^{c^{*}}+q_{2}^{c^{*}}$ in Eq (35) can be represented by $Q_{s}^{*}-Q_{c}$. Further solving the first and second-order derivatives of Eq (35) concerning *Q*_*c*_ yields:

$$\frac{\partial \prod_{g}^{c}}{\partial Q_{c}}=(p+h-o-\upsilon )+\rho [\left(e-\upsilon )(F(Q_{c})-1)-s(F(Q_{c}+q_{1}^{c}+q_{2}^{c})-F(Q_{c}+q_{1}^{c})\right)]$$ (36)

$$\frac{\partial^{2}\Pi_{g}^{c}}{\partial^{2}Q_{c}}=\rho [\left(e-\upsilon )f(Q_{c})-s(f(Q_{c}+q_{1}^{c}+q_{2}^{c})-f(Q_{c}+q_{1}^{c})\right)]>0$$ (37)

Since $\frac{\partial^{2}\Pi_{g}^{c}}{\partial^{2}Q_{c}}>0$ is constant, it shows that there exist optimal reserves $Q_{c}^{*}$ that minimize the cost to the government under this model and then associate Eqs (34–35) to obtain $Q_{c}^{*}$, as shown in Eq (38).


$$Q_{b}^{*}=F^{-1}(1-\frac{(p+h-o-\upsilon )-sB}{\rho (e-\upsilon )})$$ (38)

where $B=\frac{\left(r_{1}-r_{2}-m_{1}+m_{2})(c_{1}+r_{2}-e-s)-(r_{2}+o-m_{2}-c_{2})(r_{1}+c_{0}+s-c_{1}-\omega \right)}{\left(c_{1}+r_{2}-e-s)(r_{1}+c_{0}+s-c_{1}-\omega \right)}$.

#### 5.2.3 Supply chain coordination analysis

Likewise, coordination of the supply chain of drugs in shortage can be achieved if and only if $Q_{c}^{*}+q_{1}^{c^{*}}+q_{2}^{c^{*}}=Q_{s}^{*}$. Similarly, assuming that the demand follows a uniform distribution, the following proposition is obtained.

**Proposition 5** Under the centralized decision-making situation of government-enterprise option cooperation, the optimal government physical reserves of drugs in shortage, the optimal production capacity reserves, and the optimal API reserves of the enterprise are, respectively:

$$\{\begin{array}{l} Q_{c}^{*}=F^{-1}(1-\frac{(p+h-o-\upsilon )-sB}{\rho (e-\upsilon )})\\ q_{1}^{c^{*}}=F^{-1}[1-\frac{r_{1}-r_{2}-m_{1}+m_{2}}{\rho (r_{1}+c_{0}+s-c_{1}-r_{2})}]-Q_{c}^{*}\\ q_{2}^{c^{*}}=F^{-1}[1-\frac{r_{2}+o-m_{2}-c_{2}}{\rho (c_{1}+r_{2}-e-s)}]-Q_{c}^{*}-q_{1}^{c^{*}} \end{array}$$ (39)

Proposition 5 expresses the optimal strategy in the centralized decision-making situation of government-enterprise option cooperation. Similarly, to ensure the feasibility of the centralized decision-making model, it is necessary to ensure that the three decision variables in Eq (39) are all non-negative, whereby the following conditions are obtained: the government-enterprise can only reach the option cooperation condition when *ρ* satisfies $\rho >\frac{(p+h-o-\upsilon )-sB}{e-\upsilon }$ when *e* satisfies $\frac{\left(r_{1}+c_{0}+s-c_{1}-r_{2})(m_{2}+c_{2}-r_{2}-o\right)}{r_{1}-r_{2}-m_{1}+m_{2}}+c_{1}+r_{2}-s<e\leq M$, and when *s* satisfies $\frac{\left(r_{1}-r_{2}-m_{1}+m_{2})(M-\upsilon \right)}{c_{1}+c_{2}+h-\upsilon }-r_{1}-c_{0}+c_{1}+r_{2}<s<\omega$.

**Proposition 6** In the centralized decision-making situation of government-enterprise option cooperation, the supply chain of drugs in shortage reaches a coordinated state when $e+s=\frac{\left(M-\upsilon )(m_{2}+c_{2}-r_{2}-o\right)}{c_{1}+c_{2}+h-\upsilon }+c_{1}+r_{2}$.

Proposition 6 gives the conditions under which the supply chain reaches a coordinated state in the centralized decision-making situation. Since the government subsidy *s* and the option exercise price *e* are considered together in the centralized decision-making situation, there exists a condition that both of them are combined to satisfy the condition. Further, by solving the first-order conditions on the enterprise’s pre-storage production capacity cost *m*_1_, API production cost *c*_2_, and API stockpiling cost *m*_2_, we obtain $\frac{\partial (e+s)}{\partial m_{1}}>0$, $\frac{\partial (e+s)}{\partial c_{2}}>0$, and $\frac{\partial (e+s)}{\partial m_{2}}>0$, which shows that the sum of the government option exercise price and the substitute stockpiling subsidy increases with the increase of the enterprise’s pre-storage production capacity cost, the API production cost, and the API stockpiling cost.

**Proposition 7** Solving the first-order derivatives of the government-enterprise drug stockpile in the centralized decision-making situation concerning the drug shortage probability *ρ*, the option exercise price *e*, and the government substitute stockpile subsidy *s* yields: $\frac{\partial Q_{c}^{*}}{\partial \rho }>0$, $\frac{\partial q_{1}^{c^{*}}}{\partial \rho }<0$, $\frac{\partial q_{2}^{c^{*}}}{\partial \rho }>0$; $\frac{\partial Q_{c}^{*}}{\partial e}>0$, $\frac{\partial q_{1}^{c^{*}}}{\partial e}<0$, $\frac{\partial q_{2}^{b^{*}}}{\partial e}>0$; and $\frac{\partial Q_{c}^{*}}{\partial s}<0$, $\frac{\partial q_{1}^{c^{*}}}{\partial s}<0$, $\frac{\partial q_{2}^{c^{*}}}{\partial s}>0$.

Proposition 7 states that in the centralized decision-making situation, the government’s optimal conventional stockpile $Q_{c}^{*}$ increases with the probability of drug shortages *ρ* and the option exercise price *e*, and decreases with the increase in the government’s substitute stockpile subsidy *s*. The enterprise’s optimal production capacity stockpile $q_{1}^{c^{*}}$ decreases with the probability of drug shortages *ρ*, the option exercise price *e*, and the government’s substitute stockpile subsidy *s*. The optimal API stockpile $q_{2}^{c^{*}}$ increases with the probability of drug shortages *ρ*, the option exercise price *e*, and the government’s substitute stockpile subsidy *s*.

**Proposition 8** Based on the above results, it can be obtained that in the centralized decision-making situation of government-enterprise option cooperation, when the decision-making of government-enterprise two sides reaches equilibrium, their respective costs and profits are:

$$\begin{array}{l} \Pi_{g}^{c^{*}}=[Q_{c}^{*}(p+h)+o(q_{1}^{c^{*}}+q_{2}^{c^{*}})]-(1-\rho )\upsilon Q_{c}^{*}+\rho [\int_{0}^{Q_{c}^{*}}-\nu (Q_{c}^{*}-x)f(x)dx\\ \;+\int_{Q_{c}^{*}}^{Q_{c}^{*}+q_{1}^{c*}}e(x-Q_{c}^{*})f(x)dx+\int_{Q_{c}^{*}}^{Q_{c}^{*}+q_{1}^{c^{*}}+q_{2}^{c^{*}}}\left(e(x-Q_{c}^{*})+s(x-Q_{c}^{*}-q_{1}^{c^{*}})\right)f(x)dx\\ \;+\int_{Q_{c}^{*}+q_{1}^{c^{*}}+q_{2}^{c^{*}}}^{U}\left(e(q_{1}^{c^{*}}+q_{2}^{c^{*}})+sq_{2}^{c^{*}}+M(x-Q_{c}^{*}-q_{1}^{c^{*}}-q_{2}^{c^{*}})\right)f(x)dx] \end{array}$$ (40)

$$\begin{array}{l} \Pi_{e}^{c^{*}}=[Q_{c}^{*}(p-c_{1}-c_{2})-q_{1}^{c^{*}}(c_{2}+m_{1})-q_{2}^{c^{*}}(c_{2}+m_{2})+o(q_{1}^{c^{*}}+q_{2}^{c^{*}})]+(1-\rho )(r_{1}q_{1}^{c^{*}}+r_{2}q_{2}^{c^{*}})\\ \;+\rho [\int_{0}^{Q_{c}^{*}}\left(r_{1}q_{1}^{c^{*}}+r_{2}q_{2}^{c^{*}}\right)f(x)dx+\int_{Q_{c}^{*}}^{Q_{c}^{*}+q_{1}^{c^{*}}}\left((e-c_{0})(x-Q_{c}^{*})+r_{1}(Q_{c}^{*}+q_{1}^{c^{*}}-x)+r_{2}q_{2}^{c^{*}}\right)f(x)dx\\ \;+\int_{Q_{c}^{*}+q_{1}^{c^{*}}}^{Q_{c}^{*}+q_{1}^{c^{*}}+q_{2}^{c^{*}}}(\left(e-c_{0})q_{1}^{c^{*}}+(e+s-c_{1})(x-Q_{c}^{*}-q_{1}^{c^{*}})+r_{2}(Q_{c}^{*}+q_{1}^{c^{*}}+q_{2}^{c^{*}}-x\right))f(x)dx\\ \;+\int_{Q_{c}^{*}+q_{1}^{c^{*}}+q_{2}^{c^{*}}}^{U}\left((e-c_{0})q_{1}^{c^{*}}+(e+s-c_{1})q_{2}^{c^{*}}\right)f(x)dx] \end{array}$$ (41)

## 6 Comparison of equilibrium results

The equilibrium solutions of the government-enterprise game under the three stockpiling modes of drugs in shortage are given above and analyzed accordingly. Next, the optimal stockpiling decisions, conditions, and respective costs and profits under different situations are compared, and the following corollaries are drawn.

**Corollary 1** $Q_{b}^{*}+q_{1}^{b^{*}}+q_{2}^{b^{*}}>Q_{a}^{*}$, $Q_{c}^{*}+q_{1}^{c^{*}}+q_{2}^{c^{*}}>Q_{a}^{*}$; $Q_{b}^{*}<Q_{a}^{*}$, $Q_{c}^{*}<Q_{a}^{*}$.

From Corollary 1, it can be seen that compared with the government’s separate stockpiling model, in the government-enterprise option cooperation, both the decentralized decision-making model and centralized decision-making model increase the total amount of stockpiles of drugs in shortage and reduce the risk of government stockpiles. At the same time, this conclusion also shows that the government-enterprise option cooperation can realize a stockpile model that combines the three forms of drug physical stockpile, production capacity stockpile, and API stockpile.

**Corollary 2** $\prod_{g}^{b^{*}}<\prod_{g}^{a^{*}}$, $\Pi_{g}^{c^{*}}<\prod_{g}^{a^{*}}$; $\Pi_{m}^{b^{*}}+\Pi_{s}^{b^{*}}>\Pi_{m}^{a^{*}}+\Pi_{s}^{a^{*}}$, $\Pi_{e}^{c^{*}}>\prod_{m}^{a^{*}}+\prod_{s}^{a^{*}}$.

Corollary 2 shows that under the government-enterprise option cooperation mechanism, both decentralized and centralized decision-making, the cost of the government is reduced and the profit of the enterprises participating in the cooperation is increased.

**Corollary 3** Requirements for the probability of drug shortages under different decision scenarios for a government-enterprise to enter into option cooperation: *ρ*_*b*_<*ρ*_*c*_.

Corollary 3 shows that when the government and enterprises reach the conditions of option cooperation, the drug shortage probability requirement under the centralized decision-making model is higher than that under the decentralized decision-making model, indicating that the centralized decision-making has higher requirements for the conditions of government-enterprise option cooperation.

Given the complexity of the optimal decision-making of government under option cooperation, the following numerical calculation and sensitivity analysis give the influence of different system parameters on the optimal decision-making of government and the related cost and benefit and put forward the management revelation of practical guidance significance.

## 7 Numerical analysis and discussion

### 7.1 Numerical calculation

Based on the above model design, it is assumed that the government and two types of pharmaceutical enterprises (API and formulation independent manufacturers, and the API-F integrated manufacturer) establish cooperation on stockpiling of drugs in shortage through an option contract. According to some literature [71–74, 76] and the NMPA public data (https://www.nmpa.gov.cn/), and the Yaozhi Database (https://db.yaozh.com/), the relevant parameters are set as follows: *U* = 30000, *p* = 55, *c*_1_ = 35, *c*_2_ = 12, *c*_0_ = 44, *h* = 2, *m*_1_ = 15, *m*_2_ = 1, *r*_1_ = 10, *r*_2_ = 3, o = 8, *M* = 330, and the demand for the drug in shortage, *x*, obeys a uniform distribution on (0,*U*). Thus, it is obtained that under decentralized decision-making *ρ*_*b*_>0.187 and 7.56<*s*_*b*_<15; and under centralized decision-making *ρ*_*c*_>0.341 and 3.53<*s*_*c*_<15. It can be found that when the supply chain coordination condition is reached, the centralized decision state requires less subsidy for API substitute storage and more for drug shortage probability compared to the decentralized decision. Considering that these two variables are the influencing factors focused on in this paper, a sensitivity analysis of them is conducted next.

### 7.2 Sensitivity analysis and discussion

#### 7.2.1 Impact of variation in the probability of drug shortage

**Fig 1:**
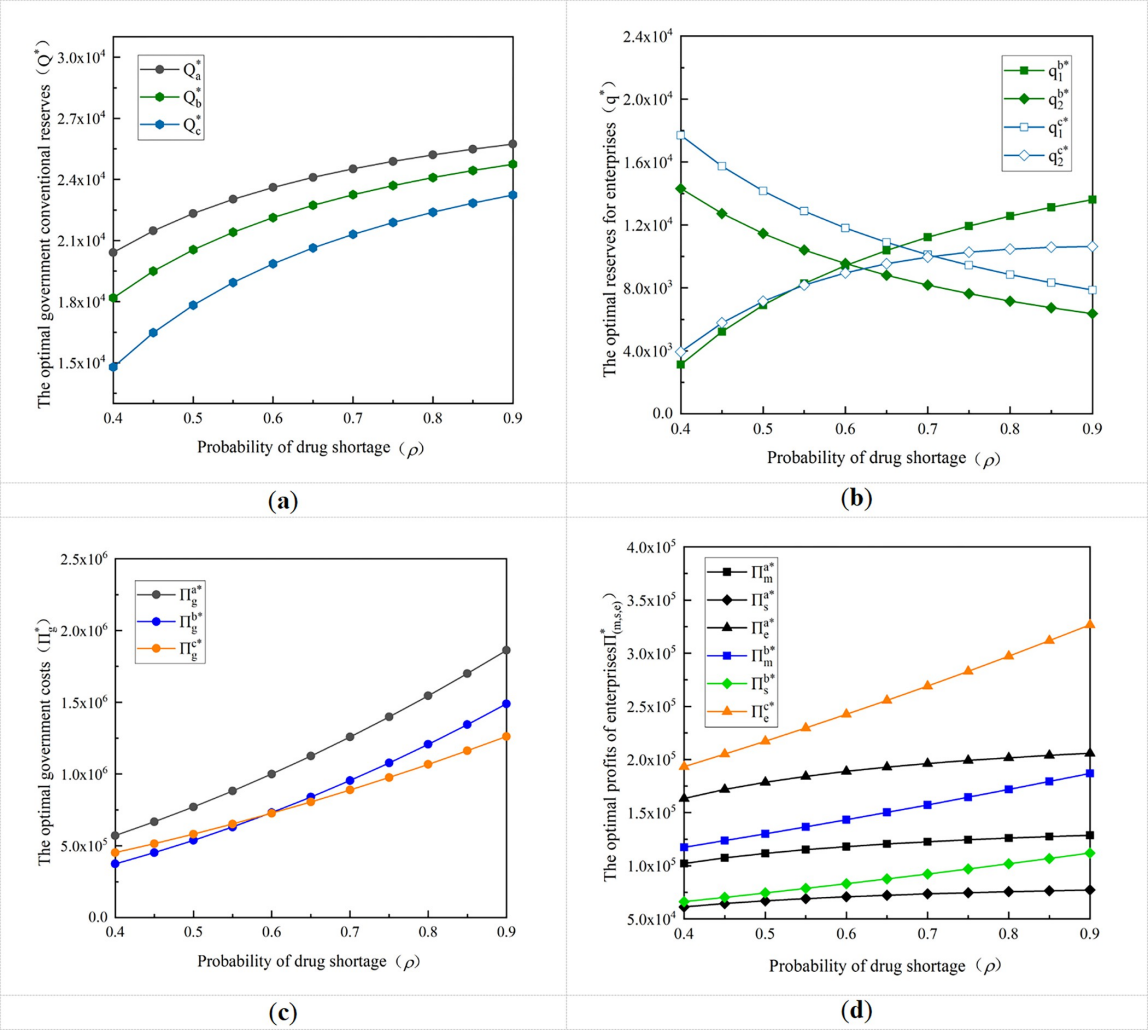
Impact of variation in the drug shortage probability on the optimal stockpiling decision of governmental enterprises and their cost and profits. **(a)** Variation of optimal government reserves with the probability of drug shortage; **(b)** Variation of the optimal reserves for enterprises with the probability of drug shortage; **(c)** Variation of government optimal cost with probability of drug shortage; **(d)** Variation of the optimal profits of enterprises with the probability of drug shortage.

To analyze the influence of the variation of drug shortage probability *ρ* on the government and enterprise stockpile decision and its costs and profits, the range of probability to satisfy the decentralized and centralized decision-making is selected, i.e., the probability of the occurrence of drug shortage is made to take the value of 0.4 and gradually increase to 0.9, and the rest of the parameters are set concerning the parameter settings in Section 7.1. Thus, the influence of *ρ* on the government’s optimal conventional reserves, the enterprise’s optimal production capacity reserves, and API reserves, as well as the costs and profits of the government and enterprises under different reserve modes is obtained, as shown in Fig 1.

Fig 1(A) and 1(B) shows that as the probability of the drug shortage grows, the optimal conventional reserves of the government increase, but the optimal conventional reserves of the government under the government-enterprise option cooperation will decrease compared with that of the government-separate stockpile model, with the most obvious decrease under the centralized decision-making of enterprises. As for enterprises, in the decentralized decision-making situation, the production capacity reserves of the formulation manufacturer will increase with the increase of the drug shortage probability, while the API reserves of the API supplier will decrease with the increase of the drug shortage probability; whereas, in the centralized decision-making situation, the production capacity reserves of the API-F integrated enterprise will decrease with the increase of the drug shortage probability, while the API reserves will increase. This is because, as the probability of a drug shortage increases, the formulation manufacturer will follow the government’s demand changes and gradually increase its production capacity reserves. And the API enterprise as a post-decision maker, taking into account the reserves of the government and the formulation manufacturer are increased, its pre-stocking of APIs will most likely not be purchased by the government, to avoid the risk of loss, it will reduce the amount of its API reserves. As for the API-F integrated enterprise, processing APIs into production capacity reserves would have increased its reserve risk, when the drug shortage probability increases, the government’s reserves increase and the enterprise meets the government’s needs while reducing its own risk of loss, it is taken to increase the amount of API reserves to reduce the amount of production capacity reserves way.

Fig 1(c) and 1(D) shows that, compared with the government-separate stockpile mode, both government costs and enterprise profits are improved under government-enterprise option cooperation, whether under centralized or decentralized decision-making, and the effect is more and more obvious with the increase of the drug shortage probability. At the same time, the trend graph of government costs with the drug shortage probability under the option cooperation also reflects that when the drug shortage probability is below a certain threshold, the government costs under decentralized decision-making are lower than those under centralized decision-making; while when the drug shortage probability is larger than the threshold, the government costs under centralized decision-making are lower than those under decentralized decision-making. This is because the enterprises under decentralized decision-making are two independent enterprises with information differences between them, and the incentive cost paid by the government will be significantly higher than that of the integrated enterprise under centralized decision-making. This suggests that for drugs that are highly susceptible to shortages, the government should prioritize cooperation with the API-formulation integrated enterprise.

#### 7.2.2 Impact of variation in the option exercise price

**Fig 2:**
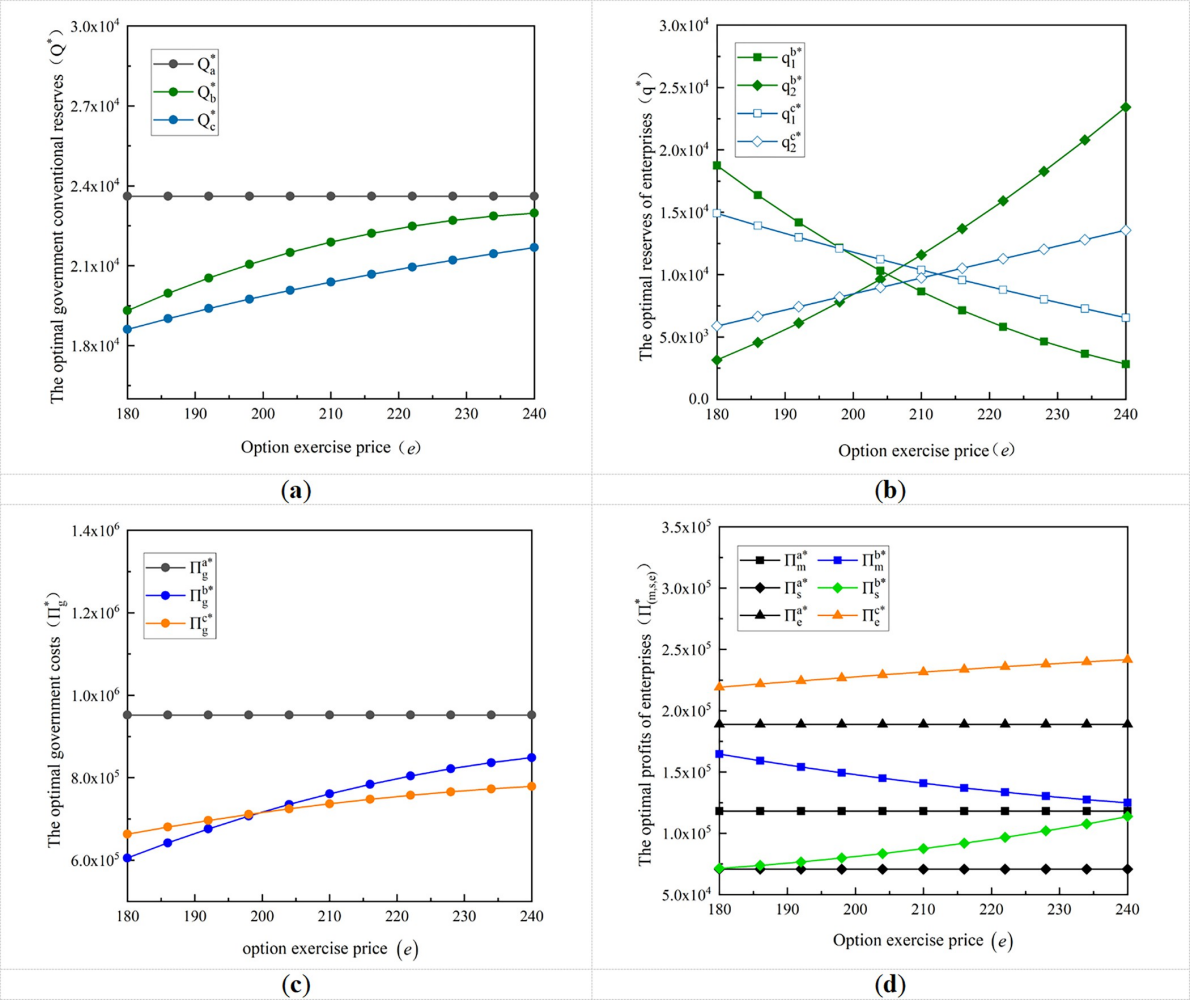
Impact of variation in option exercise price on the optimal stockpiling decision of governmental enterprises and their cost and profits. **(a)** Variation of optimal government reserves with the option exercise price; **(b)** Variation of the optimal reserves for enterprises with the option exercise price; **(c)** Variation of government optimal cost with the option exercise price; **(d)** Variation of the optimal profits of enterprises with the option exercise price.

Option exercise price is one of the important factors to motivate enterprises to participate in option cooperation, this section will analyze the impact of the variation of option exercise price *e* on the government and enterprise stockpile decision and their costs and profits. Select to meet the two decision-making modes of the option exercise price range, so that its value from 180 gradually increased to 240, and then derive the option exercise price *e* on the different stockpile mode of the government’s optimal conventional reserves, the enterprise’s optimal production capacity of reserves and the API reserves, and their costs and profits as shown in Fig 2.

According to Fig 2(A), the conventional reserves of drugs carried out by the government under option cooperation all increase with the increase of the option exercise price, and the reserves under centralized decision-making are significantly lower. This is because the ex-ante cost paid by the government is greater when the option exercise price increases and the government will gradually increase its conventional reserves based on risk and benefit considerations. Fig 2(B) shows that under decentralized decision-making, the optimal production capacity reserves of the formulation manufacturer decreases with the increase in the option exercise price, while the API reserves of the API supplier increases with the option exercise price; In the centralized decision-making situation, the optimal production capacity reserves of the API-Fintegrated enterprise decrease with the increase of the option exercise price, while the optimal API reserves increase with that price. This is mainly because when the option exercise price increases, the government, as the first decision maker, increases its reserves, and the probability of flexible purchasing from the enterprise will be reduced, leading to a decrease in the production capacity reserves of the formulation manufacturer. However, the reserves of the API supplier are affected by the production capacity reserves of the formulation manufacturer, and to fill the gap in the government’s procurement needs, the API supplier will choose to reserve more APIs for emergency procurement needs. Similarly, the same is true for the API-F integrated manufacturer.

As can be seen from Fig 2(C), as the option exercise price increases, the cost to the government under the two option cooperation models will increase but is generally lower than the cost to the government in the separate stockpile. Moreover, when the option exercise price exceeds a certain value, the government cost under centralized decision-making will be lower than that under decentralized decision-making situation. Fig 2(D) shows that enterprise profits improve in both decision situations, but the trend is different as the option exercise price increases. For the API-F integrator, profits increase with the option exercise price; for the formulation manufacturer in the decentralized decision situation, profits decrease with the option exercise price, while the opposite is true for the API supplier. This is because the API-F integrated enterprise is responsible for the balance of the two stockpiling methods, and an increase in the option exercise price by the government is a profitable behavior for it. However, for the formulation manufacturer under decentralized decision-making, when the option exercise price increases, the increase in the government’s conventional reserves leads to a decrease of its production capacity reserves, and therefore a decrease in profits; while the API enterprise, the amount of its pre-storage of APIs increases, and at the same time, when there is a flexible purchasing, the probability of emergency processing and production of finished medicines from the APIs increases, which leads to an increase in its profits.

#### 7.2.3 Impact of variation in the subsidy for substitute stockpiling of APIs

**Fig 3:**
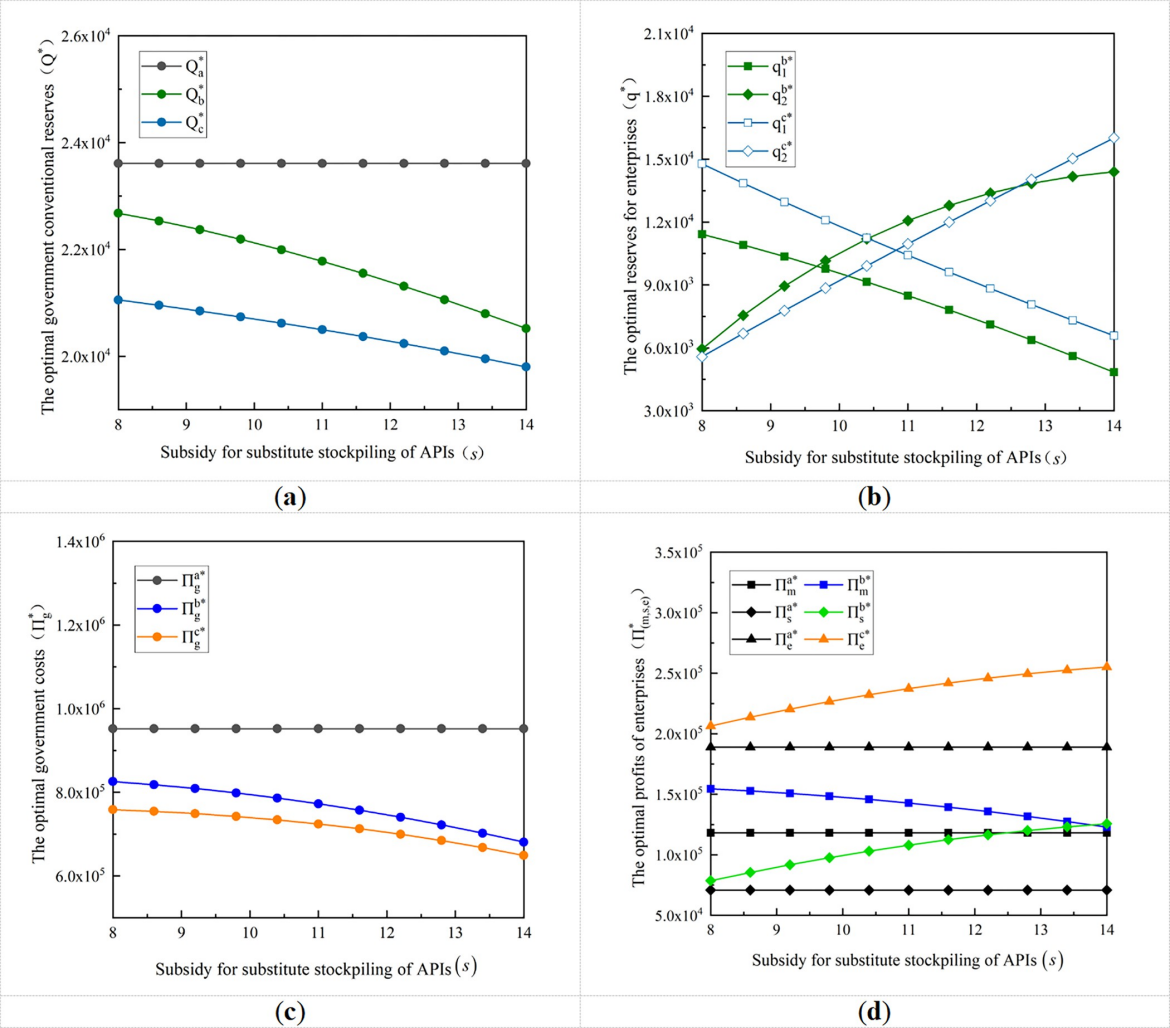
Impact of variation in subsidies for substitute stockpiling of APIs on the government-enterprise optimal stockpiling decision and their costs and benefits. **(a)** Variation of optimal government reserves with the subsidy for substitute stockpiling of APIs; **(b)** Variation of the optimal reserves for enterprises with the subsidy for substitute stockpiling of APIs; **(c)** Variation of government optimal cost with the subsidy for substitute stockpiling of APIs; **(d)** Variation of the optimal profits of enterprises with the subsidy for substitute stockpiling of APIs.

API substitute stockpile subsidy is the innovation of this study, the next step is to analyze the impact of its numerical variation on the government and enterprise stockpile decision and their costs and profits. The value of *s* is gradually increased from 8 to 14, to derive the impact of *s* on the government’s optimal conventional reserves, the enterprise’s optimal production capacity reserves, and the amount of API reserves, as well as the costs and profits of the government and enterprises under the different stockpile modes, as shown in Fig 3.

As shown in Fig 3(A), compared with the government’s separate stockpile model, the government’s conventional reserves under both government-enterprise option cooperation stockpile models increase with the increase of the substitute stockpile subsidy. It indicates that the subsidy will effectively incentivize enterprises to participate in option cooperation and reduce the pressure of government stockpiling. And it can be seen from Fig 3(B) that the government subsidy has a positive correlation effect on the enterprises to carry out API stockpiling, the more the subsidy, the more the enterprises’ API reserves. On the contrary, when the API reserves increase, the enterprise production capacity reserves will decrease. This is mainly due to the enterprise’s pursuit of profit maximization, to avoid excessive reserves caused by their own risk of loss, the formulation manufacturer to reduce production capacity reserves.

As seen in Fig 3(C), the option cooperation model can reduce the government’s stockpiling cost, and the cost reduction is more obvious when the government cooperates with the API-F integrated enterprise. Under option cooperation, the government’s costs consist of three parts, i.e., conventional purchasing costs, flexible purchasing costs, and stock-out costs. Compared to the government’s separate stockpiling model, option cooperation increases the government’s flexible procurement cost, but significantly reduces the government’s conventional procurement cost as well as the stock-out cost, which leads to a reduction in the overall cost. It can be seen from Fig 3(D) that under government-enterprise cooperation, the profits of enterprises under both decision-making modes are improved, reflecting the advantages of option cooperation. Moreover, the profit of the formulation enterprise decreases with the increase of government subsidy, the API enterprise increases with the increase of government subsidy, and the profit of the API-F integrated enterprise increases with the increase of government subsidy. The former is reduced because, the role of subsidies for storage of API is more incentives for enterprises to stockpile APIs, indirectly reducing the production capacity of the formulation manufacturer to carry out the reserve, even when the government to carry out flexible procurement needs urgent processing of raw materials for finished drugs, but also skipped the intermediate production capacity of the conversion process, so that the profits of the formulation manufacturer will be reduced with the increase in subsidies. The latter two will gain more profits due to the increase in government subsidies.

## 8 Conclusions and future prospects

### 8.1 Conclusions

Aiming at the difficulty that the first prerequisite for the reserve of production capacity of drugs in shortage is an adequate reserve of APIs, this paper established a cooperative stockpile model of options for drugs in shortage by using the theory of options contract and the theory of emergency stockpile. It incorporated the subsidy of API stockpile in the study. According to the type of production license of pharmaceutical enterprises, this paper established a decentralized decision-making model consisting of API suppliers and formulation manufacturers, and a centralized decision-making model for API-formulation integrated enterprises. The optimal reserve decisions and conditions for the government-enterprise were derived by solving the reverse-order derivation and compared with the government-alone reserve model. The study showed that:

1. The government-enterprise stockpiling model for drugs in short supply, which combines the three forms of regular government stockpiling, enterprise production capacity stockpiling, and enterprise API stockpiling, not only reduces the cost of government stockpiling but also increases the income of enterprises, which is conducive to broadening the form of drug stockpiling and improving the level of supply of drugs in short supply.
2. The probability of drug shortage during the stockpiling cycle has an important impact on the decision-making of the government and enterprises and their benefits. Because the probability of drug shortage affects the probability of flexible procurement by the government, and the level of the probability of flexible procurement determines the willingness of enterprises to participate in the cooperation of stockpiling. Enterprises are willing to cooperate with the government only when the probability of drug shortage exceeds a certain level. In addition, according to the classification of enterprise production, when the probability of drug shortage is high, priority should be given to selecting the reserved partner from the API formulation integrated production enterprises, which not only reduces the intermediate process but also reduces the cost of the government; when the probability of drug shortage is low, priority should be given to selecting the partner from the independent production enterprises of formulations and API, which reduces the risk of wastage of the reserve drugs and reduces the loss of the government and enterprises.
3. The API surrogate storage subsidy is an important incentive for enterprises to stockpile APIs. In the appropriate range of values, whether decentralized decision-making situation or a centralized decision-making situation, the optimal amount of API stockpiling increases with the increase of government subsidies. This means that, through the establishment of API storage subsidies such incentives for enterprises to participate in the reserve of APIs, improve the production capacity of drugs and physical supply capacity.

### 8.2 Managerial and social implications

We took full account of the fact that previous studies on drug shortages have rarely explored the topic of government-enterprise collaborative drug stockpiling strategies, and that government-enterprise collaborative stockpiling of emergency medical supplies under option contracts has not yet been discussed in the context of shortages, resulting in limited guidance on government-enterprise collaborative stockpiling of shortages from these studies. In this paper, the research on the government-enterprise option cooperation stockpiling strategy for drugs in shortage considering API stockpiling subsidy was aimed at providing more guarantee initiatives for the stockpiling of drugs in shortage, which has important guiding significance both at the government management level and the social level. In the following section, we discuss the application of the study and the related suggestions from the governmental management and social levels respectively.

For the governmental level: (1) Carry out diversified forms of drug stockpiling. According to the results of the study, government departments should, by the need to cope with drug shortages, carry out diversified forms of stockpiling, such as physical stockpiling, stockpiling of production capacity, and stockpiling of APIs. About production capacity reserves, it is necessary to improve foresight and targeting and to organize pharmaceutical enterprises to do a good job of stockpiling APIs in advance, to ensure that they can quickly produce the required medicines when shortages occur [29]. (2) Improving the monitoring, early warning, and information-sharing mechanism for drug shortages. In the past, scholars have suggested that the monitoring of drug shortages should be strengthened to grasp the current situation of drug shortages promptly. This paper further proposes that the information on the probability of drug shortages obtained from the monitoring data will help guide the relevant organizations to do a good job in estimating the physical and production capacity of drug reserves and reduce the risk of drug shortages. In addition, shortage monitoring and early warning information sharing should be improved to reduce the information asymmetry among drug manufacturers, distribution companies, and medical institutions, to incentivize all parties to do a good job of stockpiling drugs in advance. (3) Establishment of an incentive mechanism for stockpiling drugs in shortage. Although China is a large country for API production, the shortage of APIs has become one of the main reasons for drug shortages in China. As the research feedback, government departments should set up API stockpile subsidies and other incentives to encourage drug manufacturers to participate in API stockpiling, to improve enterprises’ drug production capacity reserves.

For the social level: (1) Strengthen the communication of information on drug shortages. Considering that China’s stockpile of drugs in shortage involves multiple stakeholders, in order to improve synergistic efficiency, it is recommended that modern technological means, such as big data, cloud computing, and blockchain, be fully utilized to set up a government-enterprise-public synergistic informatization platform to effectively transmit and communicate information on drugs in shortage [85]. (2) Encourage enterprises to scientifically assess the amount of drug reserves and the risk of supply cut-off. Enterprises involved in drug reserves are encouraged to expand their API production capacity and strengthen cooperation with multiple API enterprises. For preparation manufacturers that are unable to produce their APIs, need to communicate with government regulators promptly and utilize shortage monitoring data to make good use of their API reserves.

### 8.3 Limitations and prospects

This study closely matches the current basic conditions and actual situation of China’s stockpile of drugs in shortage, provides a basis for the establishment of a long-term cooperative relationship between the government and enterprises on drug stockpiling and provides an effective operation strategy for the good work of stockpiling drugs in shortage. Of course, there are some shortcomings in this paper. First, this paper only considers the stockpile cooperation between the government and a single pharmaceutical enterprise, and in the future, it can incorporate the discussion of the drug stockpile situation in which multiple enterprises are involved; second, this paper assumes that both the government and enterprises are completely rational, but in reality, they tend to make different decisions because of their risk preferences, and in the future, it can further incorporate the consideration of the risk preferences of the government and enterprises.
